# Inside and out

**Published:** 1891-12-05

**Authors:** 


### Passing Topics.

### INSIDE AND OUT.

The clever and indefatigable Mrs. Fawcett pursues the campaign on behalf of the emancipation of her sex without flinching, without fatigue, and, we fear, without encouragement. Man, cynical man, will not take all this declamation as seriously meant, and even if he be brought to express his sympathy for the effort, he is careful not to commit himself to a promise of support. There is much he may be willing to agree with in the abstract, possibly only with a mean desire to escape importunity, but when it comes to a practical abdication of his own superior position in the universe, and the admission of women to an equal share in its government, he feels there is a limit to complaisance.

We are much inclined to believe that Mrs. Fawcett's estimate of the dependence to be placed upon either of the two great political parties is correct. Neither of them is likely to be very much in earnest about the enfranchisement of women. Leaders, here and there, may be willing to coquet and even to feign acquiescence, but like the backsliders upon whom the vials of her wrath have been lately poured, they will easily renounce a creed to which they have never really subscribed.

Mrs. Fawcett's ingenuity has been equal to the discovery that one great reason of woman's subjection lies in her stomach. There are truths within truths, and as truth in any form is not to be gainsaid, we admit not only that the "buns and bondage" theory is a plausible one, but that it is impossible to deny the conclusion if the premises are admitted. But we go farther than this, and submit that women cannot with the due regard to accuracy which is a distinguishing feminine virtue, lay all the blame upon their insides, unless, indeed, it is contended that disorders of the digestion render them slaves not only to man but to fashion. Can it be that the morbid cravings engendered by a diet of tea and cake render woman a more easy prey to the costumier, and that in the abject obedience to the dictates of fashion at the cost too often of all that is becoming, we see the outward manifestations of a dyspeptic condition? It is not an impossible theory, and we state it for what it is worth. We know too well that cause and effect are often confounded; and it is startling to reflect that cups of tea and fashion plates may act and react on each other, but without undue speculation we may remark that as outward and visible signs will ever be regarded as interpreting much that is unseen, so man will be disposed to measure the depth and strength of the feminine mind by the distance at which she puts away from her what is comfortable and convenient in her attire. It is useless to resort to the *tu quoque* argument, and to point, for instance, to the chimney pots which are patiently borne upon the heads of the majority of men. Use is second nature, and no one can rightly deny man the merit of constancy in the matter of his dress. For our own part we have no fear that the qualities of sex will ever be obliterated or confused, or that women, however caparisoned, will be mistaken for anything but what they are. Lady Florence Dixie is of opinion that her sex is in a fair way to avenge itself upon its oppressors. We are not so sure that women object to slavery, else why welcome every fresh behest of fashion and endure cheerfully martyrdom in bodices and boots, in hoops and humps? Unless, indeed, as we have suspected, these dreadful creations are born of the nightmare which comes of impaired digestion. But then, what of the old belief that a woman dearly loves to find her master?

Agricola.

